# Risk-adapted management in stage I testicular germ-cell tumors: long-term outcomes from a single-center cohort (1994–2023)

**DOI:** 10.1007/s12094-025-04183-7

**Published:** 2026-01-17

**Authors:** Patricia Capdevila, Cristobal Carrasco, Saturnino Luján, David Ramos, Jorge Aparicio

**Affiliations:** 1 Department of Medical Oncology, Hospital Universitario y Politécnico La Fe, Avda. Abril Martorell 106, 46016 Valencia, Spain; 2 Department of Urology, Hospital Universitario y Politécnico La Fe, Valencia, Spain; 3 Department of Pathology, Hospital Universitario y Politécnico La Fe, Valencia, Spain

**Keywords:** Germ cell tumors, Risk-adapted management, Boorman’s model, Lymphovascular invasion, Rete testis invasion

## Abstract

**Background:**

Testicular cancer achieves very high cure rates, and current management aims to preserve these outcomes while minimizing treatment-related toxicity. This study aims to describe long-term outcomes of a risk-adapted program for clinical stage I (CSI) testicular germ-cell tumors (TGCT) and to evaluate histopathologic predictors of relapse.

**Methods:**

Single-center retrospective cohort (1994–2023) of CSI TGCT. Endpoints were relapse, progression-free survival (PFS), cancer-specific survival (CSS), and overall survival (OS). Associations were tested using Cox and Fisher’s exact test; model performance was assessed by discrimination with Harrell’s C-index and 5-year calibration.

**Results:**

We retrospectively analyzed 277 selected patients with TGCT, of whom 169 (61%) had CSI disease (seminoma = 104; NSGCT = 65; median age 32 years). Initial management was surveillance in 52.1% and adjuvant chemotherapy in 46.2% (carboplatin in seminoma, BEP in NSGCT). After a median follow-up of 87 months, 17 relapses occurred (10.1%). Adjuvant chemotherapy significantly reduced relapse risk (HR 0.20; *p* = 0.012). Ten-year OS and CSS were 94.4% and 99.3%, respectively. In surveillance-managed seminoma (*n* = 54), rete testis invasion independently predicted relapse (HR 9.54, 95% CI 1.29–70.3; *p* = 0.027), while the Boorman’s classification distinguished intermediate- from low-risk patients (31.8% vs 6.5%; *p* = 0.04). In NSGCT under surveillance, relapse occurred in a single patient with lymphovascular invasion (1/3, 33.3%) and in 4/31 (12.9%) without; none relapsed after adjuvant BEP.

**Conclusions:**

Risk-adapted management provides excellent long-term survival in CSI TGCT. Selective adjuvant therapy effectively prevents relapse, while histopathologic risk stratification supports individualized, deescalated strategies.

## Introduction

Testicular germ-cell tumors (TGCT) are the most common solid malignancy in young men and a paradigm of curable cancer. Around two-thirds to three-quarters present with clinical stage I disease, where long-term cancer-specific survival (CSS) routinely exceeds 98%; consequently, avoiding overtreatment and late toxicities is a central goal of care [1, 2].

After orchiectomy, active surveillance is the preferred strategy for most stage I seminoma patients, with adjuvant carboplatin considered for selected higher risk profiles; routine radiotherapy has largely been abandoned [1]. In stage I nonseminoma (NSGCT), surveillance and adjuvant BEP are standards, while primary RPLND is reserved for selected cases and specific settings [3].

In clinical stage I seminoma, tumor size > 4 cm and rete testis invasion (RTI) have traditionally been accepted as prognostic markers for relapse and underpinned early risk-adapted strategies [1, 4, 5]. However, systematic reviews and contemporary guidelines emphasize that the underlying evidence is heterogeneous and insufficient to mandate routine adjuvant therapy solely on these factors [6, 7]. More recently, an individual-patient-data analysis by the EAU Testicular Cancer Guidelines Panel [8] proposed a model integrating tumor size, RTI, and LVI that outperformed the traditional > 4 cm/RTI schema, stratifying 5-year relapse risk and identifying subgroups with rates up to 44% when multiple factors are present. Notably, the prognostic impact of LVI has been supported by central pathology reviews and robust multicenter validation studies, reinforcing its clinical relevance [9, 10]. Current European guidance acknowledges these prognostic factors but still cautions against risk-factor-driven adjuvant therapy in all-comers, supporting active surveillance as the preferred approach for most CSI seminoma patients [11].

In NSGCT, lymphovascular invasion (LVI) remains the most consistently validated predictor of relapse, identifying patients with an estimated recurrence risk of over 50% under surveillance when present; by contrast, relapse risk is typically below 20% when LVI is absent [11–13]. The Spanish Germ Cell Cancer Group (SGCCG) has demonstrated the feasibility of risk-adapted strategies in multicenter cohorts—particularly in seminoma—supporting surveillance in low-risk profiles and short-course adjuvant therapy in higher risk settings [1, 14]. This risk-adapted approach has likewise been validated in stage I NSGCT, with excellent control using selective adjuvant therapy guided by pathological risk factors [15]. However, real-world validation of newer stratification tools (e.g., the Boorman’s framework) in contemporary datasets with prolonged follow-up remains limited. Imaging and follow-up have also evolved toward radiation-sparing paradigms (e.g., MRI-based or reduced-intensity CT schedules) [16], which may influence the timing and mode of relapse detection and require a modern interpretation of legacy series.

Against this background, single-center longitudinal datasets provide granular insight into how risk-adapted management performs across practice eras. Building on the SGCCG tradition and aligned with European guidance, we report long-term outcomes from a 1994–2023 institutional cohort of stage I TGCT: (i) adoption of risk-adapted strategies over time; (ii) relapse, PFS, CSS, and OS by histology and initial management; and (iii) prognostic performance of established clinico-pathologic factors and the Boorman’s framework in real-world practice.

## Methods

We performed a retrospective cohort study, including all consecutive patients diagnosed with TGCT between 1994 and 2023 in our center. After prespecified exclusions—such as extragonadal primaries, nongerm-cell histologies, and incomplete records—only gonadal TGCT were retained. The present analysis focused on patients with clinical stage I disease after orchiectomy. Histological classification followed the 2016 WHO criteria, and clinical and pathological data were obtained from medical records, including age, histology (seminoma vs NSGCT), tumor size, RTI and LVI. Unknown values were treated as missing (NA) and excluded from the corresponding analyses without imputation.

Post-orchiectomy management was categorized as active surveillance, adjuvant chemotherapy (carboplatin in seminoma, BEP in NSGCT), or radiotherapy (seminoma only, limited to the early study period). Risk-adapted management was defined according to established histopathologic criteria, namely tumor size > 4 cm and/or RTI for seminoma and LVI for NSGCT. In stage I seminoma, adjuvant carboplatin was offered to patients meeting one or both classical risk factors according to SGCCG risk-adapted criteria [1, 5]. Patients were recommended to have risk-adapted therapy unless they preferred (and opted for) any option. Concordance was considered present if low-risk patients underwent surveillance, while high-risk patients received adjuvant therapy. In the first two decades of the study, patients received two courses of adjuvant carboplatin or BEP, whereas only one course of either chemotherapy was employed in the last decade.

The primary endpoint was progression-free survival (PFS), defined as the interval from orchiectomy to relapse, progression, or last follow-up. Overall survival (OS) and CSS were analyzed as secondary outcomes. In the seminoma subgroup managed with surveillance, prognostic modeling was conducted both for individual risk factors and according to the Boorman’s three-factor model, which integrates categorical tumor size (≤ 2, > 2–5, > 5 cm), RTI, and LVI to define low-, intermediate-, and high-risk groups. Discriminative performance was assessed with Harrell’s C-index and calibration with bootstrap resampling (B = 200).

Continuous variables were summarized as medians with ranges and categorical variables as frequencies and percentages. Between-group comparisons used χ^2^ or Fisher’s exact tests. Survival outcomes were estimated with the Kaplan–Meier method and compared by the log-rank test, while hazard ratios (HR) and 95% confidence intervals (CI) were obtained from Cox proportional hazards models. Exact 95% CIs for proportions were derived using the Clopper–Pearson method. All analyses were performed using R software (version 4.4.0). Statistical significance was set at a two-sided p < 0.05.

The study was approved by the Clinical Research Ethics Committee of our center and conducted in accordance with the Declaration of Helsinki.

## Results

Between 1994 and 2023, 277 selected gonadal TGCT were treated at our center, of which 169 (61%) had clinical stage I and constituted the analytic cohort (seminoma n = 104; NSGCT n = 65). Median age at diagnosis was 32 years (16–63). Baseline pathology (tumor size, RTI, LVI) and initial post-orchiectomy management are summarized in Table 1.

**Table 1:** Baseline characteristics of the clinical stage I cohort (1994–2023)

Characteristic	Overall (*n* = 169)	Seminoma (*n* = 104)	NSGCT (*n* = 65)
Age at diagnosis, median (range), y	32 (16–63)	35 (20–63)	30 (16–56)
Histologic-specific prognostic factors			
> 4 cm (seminoma)		51 (49.0)	–
RTI present (seminoma)		26 (25.0)	–
LVI present (seminoma)		18 (17.3)	–
LVI present (NSGCT)		–	35 (53.8)
Initial management			
Surveillance, *n* (%)	88 (52.1)	54 (51.9)	34 (52.3)
Adjuvant chemotherapy, *n* (%)	78 (46.2)	47 (45.2)	31 (47.7)
Adjuvant radiotherapy, *n* (%)	3 (1.8)	3 (2.9)	0 (0.0)

Data are *n* (%) unless indicated; age is median (range). Missing/unknown values were treated as NA and excluded from percentage calculations

*TGCT* testicular germ-cell tumor; *NSGCT* nonseminomatous germ-cell tumor; *RTI* rete testis invasion; *LVI* lymphovascular invasion

We analyzed relapse incidence according to prognostic factors and initial post-orchiectomy management, stratified by histology (Table 2). In seminoma (n = 102 evaluable for this analysis), patients with ≥ 1 risk factor—RTI and/or tumor size > 4 cm (n = 55)—had 7/16 (43.8%) relapses under surveillance versus 1/39 (2.6%) after adjuvant chemotherapy (Fisher p = 0.00038). Among patients without risk factors (n = 47), relapses were 2/37 (5.4%) on surveillance and 1/10 (10.0%) after adjuvant therapy. In NSGCT (n = 65), using LVI as the sole prognostic factor, LVI-positive patients had 1/3 (33.3%) relapses on surveillance and 0/19 (0.0%) after adjuvant BEP; among LVI-negative patients, relapses were 4/31 (12.9%) on surveillance and 0/12 (0.0%) after adjuvant chemotherapy.

**Table 2:** Relapse rates by histology, risk group, and initial management, with p values for associations between risk factors and (i) adjuvant therapy use and (ii) relapse

Histology	Risk group	Adjuvant treatment association	Treatment	Patients (*n*)	Relapses (*n*)	Relapse rate (%)	Association with relapse
Seminoma	≥ 1 factor (RTI and/or > 4 cm)	RTI: *p* < 0.001 Size: *p* < 0.05	Surveillance	16	7	43.8	Size, RTI: *p* = 1.0
			Adjuvant CT	39	1	2.6	
	No risk factors	< 0.001	Surveillance	37	2	5.4	
			Adjuvant CT	10	1	10.0	
NSGCT	LVI present	< 0.001	Surveillance	3	1	33.3	LVI: *p* = 0.65
			Adjuvant CT	19	0	0.0	
	LVI absent	< 0.001	Surveillance	31	4	12.9	
			Adjuvant CT	12	0	0.0	

Risk groups were defined as tumor size ≥ 4 cm and/or RTI for seminoma, and LVI for NSGCT. Values are *n* (patients) and relapse rate (%); tests by Chi-square or Fisher’s exact, as appropriate

Across histology-specific subgroups, classical risk factors (seminoma: size ≥ 4 cm, RTI; NSGCT: LVI) were not significantly associated with PFS. Five-year PFS remained high and similar in seminoma patients with and without risk factors (p = 0.30), and LVI did not translate into a higher progression risk in NSGCT (p = 0.40). Notably, these factors were strongly associated with the indication for adjuvant therapy (all p < 0.001), indicating that treatment decisions closely followed the risk-adapted strategy. Detailed results of these associations are provided in Table 2.

Among seminoma patients receiving adjuvant carboplatin, 21/48 (43.8%) received one cycle and 27/48 (56.3%) two cycles. Two cycles were predominantly used in earlier periods—13/14 (92.9%) in 1994–2003 and 9/9 (100.0%) in 2004–2013—whereas a shift toward single-cycle regimens occurred from 2014 onward, with 20/25 (80.0%) receiving one cycle. Relapse rates were low and comparable between the one- and two-cycle groups (4.8% vs 3.7%). In stage I NSGCT managed with adjuvant therapy, single-cycle BEP became standard in 2014–2023 (n = 13), with no relapses observed; earlier periods predominantly used two cycles (mostly BEP), also with 0 relapses.

Within the NSGCT subgroup, the impact of embryonal carcinoma (EC) predominance on treatment allocation and relapse was further explored. Among 65 patients with stage I NSGCT, 10 (15.4%) had pure embryonal carcinoma (EC) and 55 (78.5%) had mixed histology. Of the mixed tumors, 51 contained an EC component and 4 showed no EC elements. Quantitative data on EC proportion were available in 28 (54.9%) cases, with 16 (57.1%) showing ≥ 50% EC and 12 (42.9%) < 50%; the EC proportion was unknown in 23 (45.1%) cases.

Adjuvant chemotherapy was more frequently administered in patients with pure EC (7/10, 70.0%) or mixed tumors containing ≥ 50% EC (13/16, 81.3%) compared with those with < 50% (7/12, 58.3%) or unknown EC proportion (2/23, 8.7%) (*p* < 0.001). Relapses were uncommon across all subgroups, with none observed among patients with pure EC or mixed tumors containing ≥ 50.0% EC. Only one recurrence occurred in the < 50.0% group and four among cases with unknown EC proportion. No significant association was found between EC proportion and relapse occurrence (*p* = 0.22).

**Fig. 1:**
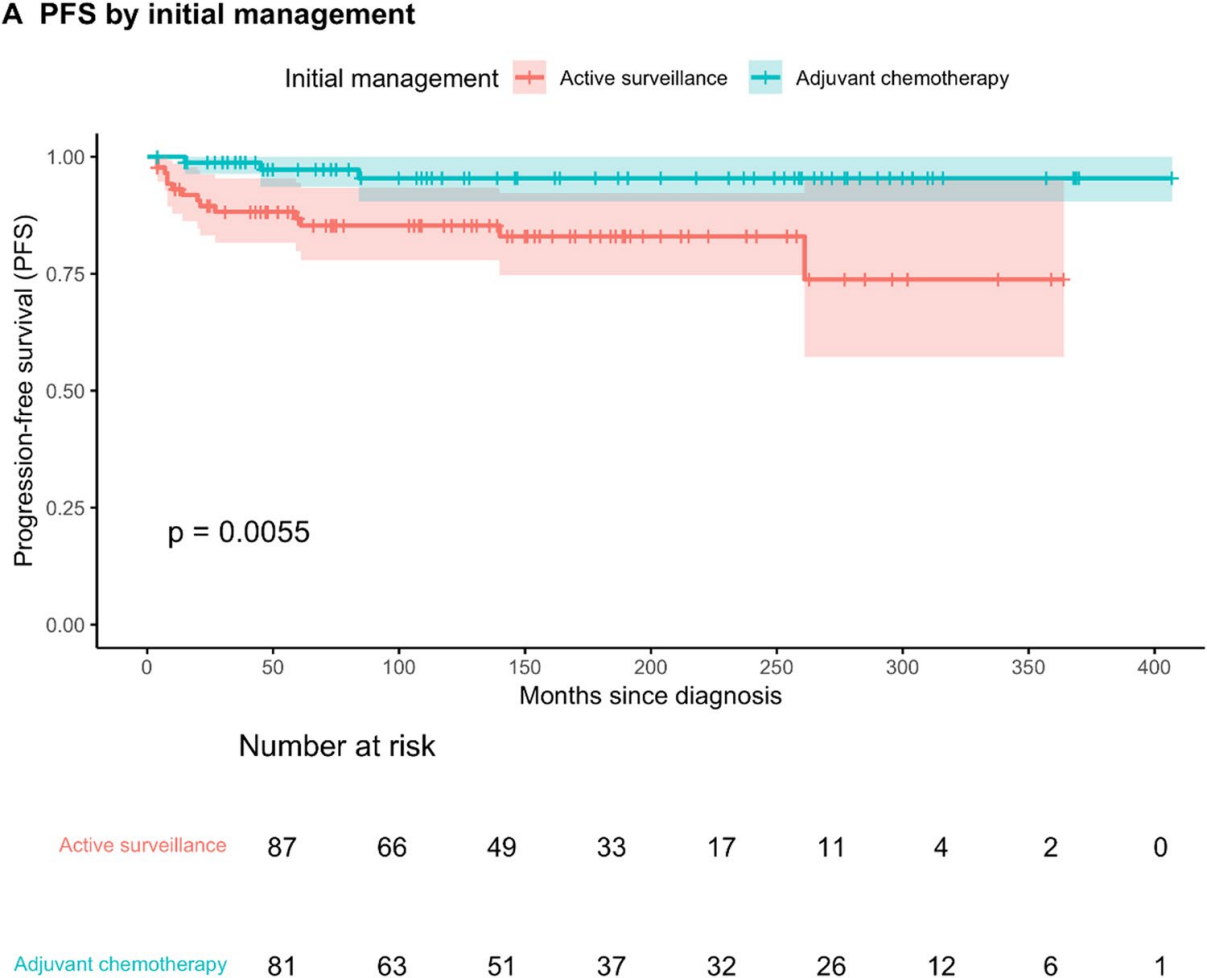
Kaplan–Meier curves for PFS according to initial management (active surveillance vs adjuvant chemotherapy), *p* < 0.05

**Fig. 2:**
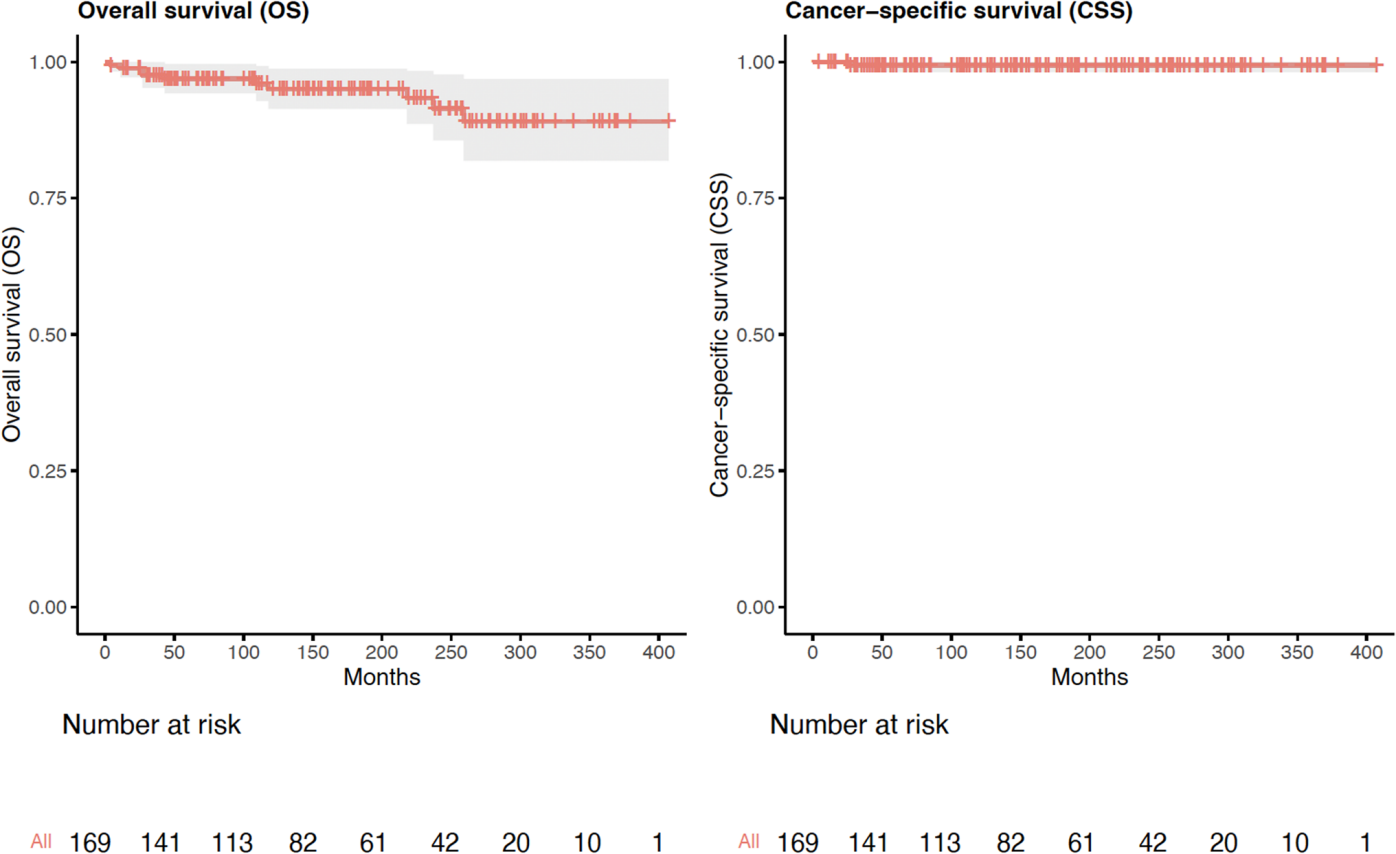
Overall survival (OS) and cancer-specific survival (CSS) in the clinical stage I cohort. Survival curves were estimated using the Kaplan–Meier method

After a median follow-up of 87 months (13–326), 17 relapses occurred (10.1%). In univariable Cox analysis, adjuvant chemotherapy was associated with a significantly lower risk of progression compared with active surveillance (HR 0.20, 95% CI 0.06–0.71; p = 0.012); the association was consistent by Wald (p = 0.010) and log-rank (p = 0.006) tests, with a model concordance index of 0.667 (Fig. 1). Among stage I patients, 75.0% of relapses occurred within 24 months (median 17 months) and were predominantly retroperitoneal (70.0%). Ten-year OS and CSS were 94.4% and 99.3%, respectively (Fig. 2).

Classical risk factors were then evaluated in relation to PFS. In the whole cohort (treated and surveilled), size ≥ 4 cm and RTI (seminoma) and LVI (NSGCT) were not significantly associated with PFS, a finding likely explained by the preferential use of adjuvant therapy in patients with adverse features. Restricting to surveillance-only patients (n = 88), prognostic modeling was performed separately by histology. In seminoma (n = 54), RTI emerged as a significant predictor of relapse (HR 9.54; 95% CI 1.29–70.3; p = 0.027), tumor size > 4 cm showed a non-significant trend (HR 4.28; 95% CI 0.71–25.8; p = 0.11), and LVI was not associated (22.2% vs 15.9%; p = 0.64). When adverse features were combined (any of size > 4 cm, RTI, or LVI), relapse occurred in 29.2% versus 6.9% in those without risk factors (Fisher p = 0.06). In the NSGCT surveillance subgroup (n = 34), three patients were LVI-positive and 31 were LVI-negative; relapses were 1/3 (33.3%) vs 4/31 (12.9%) (Fisher p = 0.39). No relapses occurred after adjuvant BEP.

**Fig. 3:**
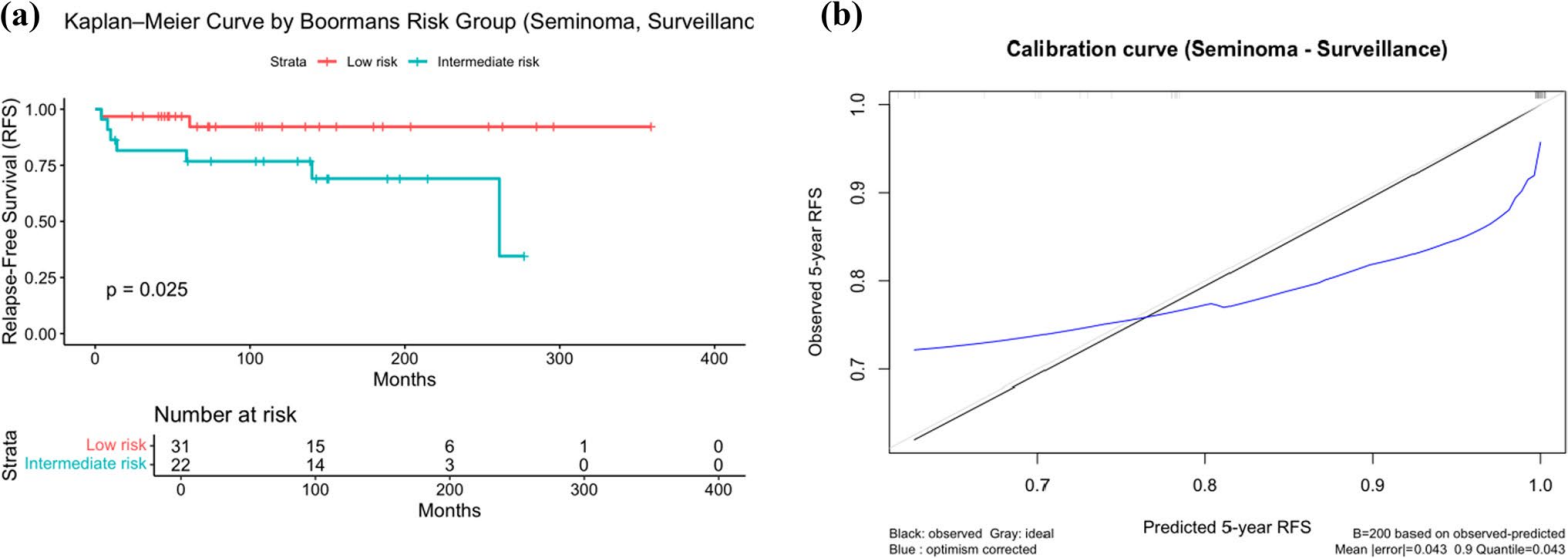
Performance of the Boorman’s three-factor model in clinical stage I seminoma under active surveillance. **a** Kaplan–Meier relapse-free survival (RFS) by risk group (low vs intermediate); numbers at risk shown. Log-rank *p* = 0.025. **b** Calibration plot for 5-year RFS predictions. Gray line = ideal; black = observed; blue = optimism-corrected (bootstrap B = 200). Mean |error|= 0.043

Given these signals in surveillance-only seminoma, we applied the Boorman’s three-factor model (categorical size ≤ 2, > 2–5, > 5 cm; RTI; LVI). Among seminoma patients managed with active surveillance (n = 54), Boorman’s risk classification was available in 53 owing to one missing component. No patient met high-risk criteria. Intermediate-risk (n = 22) had more relapses than low-risk (n = 31) (31.8% vs 6.5%; OR 6.51, 95% CI 1.07–72.0; p = 0.025, Fisher; Fig. 3a). In time-to-event analysis, intermediate versus low risk showed HR 5.04 (95% CI 1.04–24.4; p = 0.044), with moderate discrimination (Harrell’s C-index 0.673). The 5-year cumulative incidence of relapse was 23.2% (95% CI 3.1–39.2) versus 3.2% (0.0–9.2), an absolute difference of 20.0 percentage points. Five-year calibration showed acceptable agreement between predicted and observed PFS; the bootstrap optimism-corrected curve (B = 200) yielded a mean absolute calibration error of 0.043 (90th percentile 0.043), with minor mid-range miscalibration and close alignment at higher predicted PFS (Fig. 3b).

## Discussion

Our overall relapse incidence of 10.1% with long follow-up (median 87 months) is consistent with risk-adapted practice across both histologies. In seminoma, the higher crude relapse proportion (11.5%) versus SGCCG (16/227, 7.0%) and SWENOTECA series (69/897, 7.7%) [1, 5, 16, 17] is plausibly explained by longer follow-up, a higher prevalence of adverse features, and a more conservative use of adjuvant carboplatin in favor of surveillance, together with the temporal shift from two to one cycle [14, 17]. By contrast, NSGCT relapse (7.7%) approximates benchmarks from risk-adapted programs; across series, 5-year relapse is generally in single digits and CSS approaches 99–100% [1, 5, 12, 17–19].

Crucially, the strong association between pathological risk factors and treatment allocation confirms appropriate implementation of a risk-adapted approach in our cohort. In seminoma, adverse features were preferentially treated with adjuvant carboplatin; in NSGCT, LVI drove the indication for adjuvant BEP, with no relapses after treatment. Within NSGCT, adjuvant therapy was also used more often in pure EC or mixed tumors with ≥ 50% EC—again with absence of relapse after adjuvant treatment—whereas EC proportion itself did not predict relapse under surveillance [3, 11, 23].

Evidence on classical seminoma predictors (tumor size, RTI) is heterogeneous across systematic reviews [6, 7]. In surveillance cohorts, 5-year relapse typically ranges 13–18% with CSS 99% [4, 19–21]; in our surveillance-only subset, it was 8/54 (14.8%), within that range. In the whole cohort, size ≥ 4 cm and RTI were not associated with PFS—consistent with treatment selection [1, 5, 11]. In the surveillance-only subset, the expected gradient reemerged: RTI independently predicted relapse (HR 9.54; 95% CI 1.29–70.3; p = 0.027), tumor size ≥ 4 cm showed a non-significant trend, and LVI was not prognostic. These findings are consistent with prior pooled and systematic analyses [4, 6, 7] and with large surveillance programs [19–21], with conditional risk decreasing over time among patients remaining event-free [22]. The Boorman’s three-factor model showed moderate discrimination and acceptable 5-year calibration, supporting risk counseling in routine care [8, 9].

For NSGCT, evidence consistently identifies LVI as the dominant predictor of relapse under surveillance [18, 23–25], and a single BEP cycle reduces relapse to about 3% in risk-adapted programs [26]. In our surveillance subset, the LVI–relapse association was attenuated—likely reflecting small numbers and preferential allocation of LVI-positive patients to adjuvant chemotherapy—while no relapses occurred after adjuvant BEP.

Finally, adjuvant practice deescalated from two to one cycle with comparable relapse rates, supporting single-cycle regimens when adjuvant therapy is chosen. Since 2014, single-cycle adjuvant therapy has predominated in both seminoma (carboplatin) and NSGCT (BEP), with no NSGCT relapses and similarly low relapse rates after one versus two cycles in seminoma.

This study is limited by its retrospective, single-center design and few relapse events—particularly in NSGCT surveillance—while strengths are long follow-up, clear capture of management eras, and consistent real-world application of a risk-adapted pathway.

Overall, surveillance remains the backbone for most CSI TGCT, and selective adjuvant therapy effectively reduces relapse risk in patients with adverse features. The Boorman’s three-factor model reproduced its discrimination and acceptable calibration in our cohort, making it a useful aid for risk counseling and shared decision-making. Prospective studies in unselected cohorts should further test high-risk strata and refine follow-up intensity.

## Data Availability

Data underlying the findings are available from the corresponding author on reasonable request, subject to institutional policies and data-protection regulations.
